# Close of Volume Sixth—Third Series

**Published:** 1850-12

**Authors:** 


### THE WESTERN JOURNAL.

THIRD SERIES.—VOL. VI.—NO. VI.

LOUISVILLE, DECEMBER 1, 1850.

### CLOSE OF VOLUME SIXTH—THIRD SERIES.

This number closes the sixth volume, of the third series of the Western Journal of Medicine and Surgery, and we cannot permit the occasion to pass, without calling the attention of the readers to the important subject of medical periodical literature. For the advancement of the interests of the medical profession an active periodical literature is absolutely essential. We know of no department of science whose interests are more deeply interested in that kind of intercourse. The universal fields of nature are store-houses of facts for the physician, and in proportion to the universality of the experience of physicians among the races of men, that may be acquired by the practitioner of medicine, in that proportion is he qualified to discharge his multiform, arduous, ever-varying, and onorous duties. The busy pages of the medical periodical press are the only source that can convey the information thus needed. They gather into one focus the rays of light scattered over the medical world, they reveal the lights, experience, and observation of all the leading practitioners, in all the varieties of human existence. The medical philosopher and the experienced practitioner meet upon these pages, and give their combined influence towards the advancement of the whole profession, and the cure of disease.

These important considerations plead loudly for the support of medical periodicals. The success of the periodical is a joint concern, in which the interests of the readers, editors, and publishers are involved. The reader finds his dividend in the extension of his usefulness as a practitioner of medidine; the editors find theirs in the amount of service they may render to the cause of medical science; and the publishers look for theirs in the remuneration given for their labor, their heavy expenses, and the wear and tear of their material. None but those who have tried it can form any just conception of the severity of the labors that devolve upon the conscientious editor of a medical journal. To be able to fulfil his duties to his readers, he must be a practitioner of medicine and surgery; in connection with the demands of practice, he must read all the medical journals that he can command; he must read all manuscripts presented for publication, and must often write a great deal himself; he must keep pace with the new medical books, constantly issuing from the press, and pass his judgement upon them. These are the mental operations of an editor; the drudgery we shall not detail.

We have endeavored, to the best of our ability, to keep the Western Journal of Medicine and Surgery up to the points of excellence we have indicated. How far we have succeeded in our efforts is left to the judgement of the readers of the work. We are about to commence a new volume, and while we desire to make it more useful than its immediate predecessor, we shall be careful not to let it fall behind what a journal devoted to practical medicine should be.

We appeal to the readers of the Western Journal to extend its usefulness, and not only by enlarging its list of subscribers, but by communications of an instructive character to the profession. By this means, they will be useful to themselves as well as to their medical brethren. We pray the attention of the readers of this journal to these requests.

